# Alcohol-related peripheral neuropathy: a systematic review and meta-analysis

**DOI:** 10.1007/s00415-018-9123-1

**Published:** 2018-11-22

**Authors:** Thomas Julian, Nicholas Glascow, Rubiya Syeed, Panagiotis Zis

**Affiliations:** 1 The Medical School, University of Sheffield, Beech Hill Rd, Sheffield, S10 2RX UK; 2 Academic Department of Neurosciences, Sheffield Teaching Hospitals NHS Trust, Sheffield, UK; 3 Medical School, University of Cyprus, Nicosia, Cyprus

**Keywords:** Alcohol, Alcoholic, Neuropathy, Ethanol, Polyneuropathy

## Abstract

The primary aim of this systematic review was to establish the prevalence, character, and risk factors of peripheral neuropathy amongst chronic alcohol abusers and to identify the most appropriate management strategies. In this review, possible pathogenetic mechanisms are also discussed. A systematic, computer-based search was conducted using the PubMed database. Data regarding the above parameters were extracted. 87 articles were included in this review, 29 case–control studies, 52 prospective/retrospective cohort studies and 2 randomised control trials, 1 cross sectional study, and 3 population-based studies. The prevalence of peripheral neuropathy amongst chronic alcohol abusers is 46.3% (CI 35.7– 57.3%) when confirmed via nerve conduction studies. Alcohol-related peripheral neuropathy generally presents as a progressive, predominantly sensory axonal length-dependent neuropathy. The most important risk factor for alcohol-related peripheral neuropathy is the total lifetime dose of ethanol, although other risk factors have been identified including genetic, male gender, and type of alcohol consumed. At present, it is unclear what the pathogenetic mechanisms for the development of neuropathy amongst those who chronically abuse alcohol are, and therefore, it is unknown whether it is attributed to the direct toxic effects of ethanol or another currently unidentified factor. There is presently sparse data to support a particular management strategy in alcohol-related peripheral neuropathy, but the limited data available appears to support the use of vitamin supplementation, particularly of B-vitamin regimens inclusive of thiamine.

## Introduction

Alcohol abuse is known to cause a range of neurological disorders, including cerebellar ataxia, confusion, cognitive impairment, and peripheral neuropathy [1]. Neuropathy associated with chronic alcohol abuse may involve large and/or small (including autonomic) fibres and is rather heterogeneous in its clinicopathological features [2, 3]. The earliest known description of neuropathic symptoms associated with ingestion of alcohol were noted by Lettsom in 1787, describing the presentation of paralysis and hypoesthesia which was of greater prominence in the legs than the arms [4]. Presently, peripheral neuropathy amongst chronic alcohol abusers remains an entity of disputed character and pathogenesis. Its current obscurity is likely attributable to the complex range of physiological derangements that come with chronic alcohol abuse—many of which have the capacity to cause neuropathy. Some of the factors discussed in literature which can attribute to the neuropathy presenting in these patients are the direct toxicity of alcohol, nutritional deficiencies (particularly thiamine and B12), hepatic cirrhosis, impurities of alcoholic beverages (for instance, lead) and deranged blood glucose [3, 5, 6]. The interaction of these factors has not only complicated discerning the most important pathological mechanisms of neuropathy in alcohol abuse, but also prevented characterisation of the typical features as the various elements affect the nervous system differently. Although alcoholic toxicity is not firmly established as the only pathogenic factor in neuropathy amongst alcohol abusers, in this review the entity shall be referred to as “alcohol-related neuropathy”.

The aim of this systematic review is to characterise the presentation of alcohol-related peripheral neuropathy, to determine the typical ancillary test results, to establish the importance of various risk factors and to explore the likely pathogenetic mechanisms. Due to the breadth of the literature surrounding this topic, this review shall focus exclusively upon peripheral neuropathy, without discussing autonomic neuropathy.

## Methods

### Protocol registration

This review was prospectively registered to PROSPERO. The registration number for this review is CRD42018099910.

### Literature search strategy

A systematic search was performed on 10.06.18 using the PubMed database. For the search, two medical subject heading (MeSH) terms were used. Term A was “alcohol OR alcoholic OR ethanol”. Term B was “neuropathy OR polyneuropathy”. Human subject and English language filters were applied in our search. The reference lists of included articles were scanned for further articles which may fall within the scope of this review and were included where appropriate.

### Inclusion and exclusion criteria

Articles eligible to be included in this review were required to meet the following criteria:

1. The article discussed peripheral neuropathy related to chronic alcohol consumption. Neuropathy was diagnosed clinically and/or with nerve conduction studies (NCS).
2. The study was conducted using human subjects.
3. The article was written in English language.

Articles meeting the following criteria were excluded from our review:

1. Case reports.
2. Non-original articles (i.e., review articles, letters to the Editor, expert opinion papers etc.).
3. Animal studies.
4. Duplicate articles (identical publications).
5. Studies of patient populations with other causes of neuropathy or with comorbidities which may cause neuropathy (e.g., diabetes, vasculitis, mechanical trauma).
6. Studies exclusively detailing other forms of neuropathy, such as autonomic or optic neuropathy.
7. Articles which could not be obtained despite University inter library request, British Library request and attempts made to contact the article author.
8. Studies which contained subjects who had consumed any forms of alcohol other than ethanol (e.g., methanol) or who consumed illegally manufactured or homemade alcohol as this is likely to contain impurities.
9. Articles referring to description of neurophysiological techniques not specific to alcohol-related neuropathy.
10. Articles detailing pilot treatments that have not been replicated or further confirmed with larger studies.

All articles were abstract screened by a minimum of three authors in a blinded fashion using Rayyan software to ensure accuracy. Those found to meet any of the exclusion criteria were removed and any conflicts were settled by consensus during a face-to-face meeting in which the abstracts were reread. All remaining papers were screened again as a full article by at least two authors and conflicts were settled as before. Where a paper was not available online, University interlibrary request was made for the item, British Library request and failing these we attempted to find the authors contact details.

### Data collection process

Data were extracted from each study in a structured coding scheme using Google Sheets and included: Population size; demographics; total alcohol consumption and duration of alcohol consumption; prevalence of alcohol-related neuropathy; clinical and neurophysiological data; prognosis; biopsy/necropsy results; nutritional status; management; pathogenesis; and risk factors. Where there was uncertainty with respect to how data should be interpreted, at least three authors discussed the paper to ensure consensus.

### Synthesis of results

This study used aggregate data where ever possible. Frequencies and descriptive characteristics extracted were calculated. This study is reported in accordance with the preferred reporting items for systematic reviews and meta-analysis (PRISMA) guidelines [7]. Statistical calculation of pooled proportions was conducted in R language, using the default settings of the “meta” package and the “metaprop” function with a random effects model [8].

### Assessment of bias

Randomised control trials were assessed for bias using JADAD scoring [9].

### Compliance with ethical guidelines

This article is based upon previously published studies. There are, therefore, no ethical concerns with regard to this study.

## Results

### Study characteristics

The literature search heralded 668 results. In total, 585 papers did not meet the inclusion/exclusion criteria and were excluded. By scanning the reference lists of included studies, an additional 4 papers were identified. Therefore, a total of 87 articles were included in this review.

This included 29 case–control studies, 52 prospective/retrospective cohort studies and 2 randomised control trials, 1 cross sectional study, and 3 population-based studies.

**Fig. 1:**
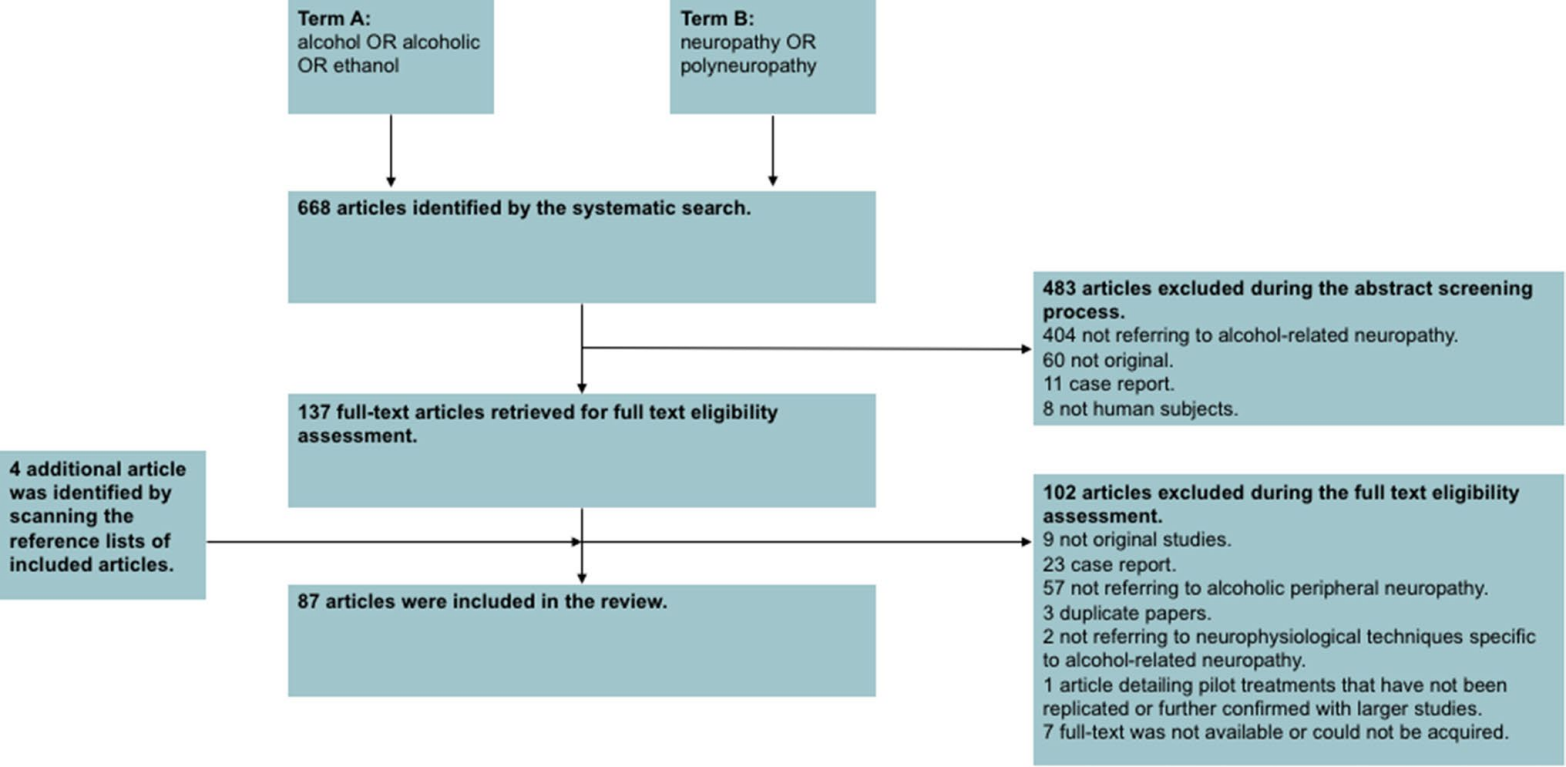
PRISMA chart detailing the inclusion/exclusion process

The study selection process is illustrated in Fig. 1.

### Prevalence

#### Prevalence of peripheral neuropathy amongst chronic alcohol abusers

**Fig. 2:**
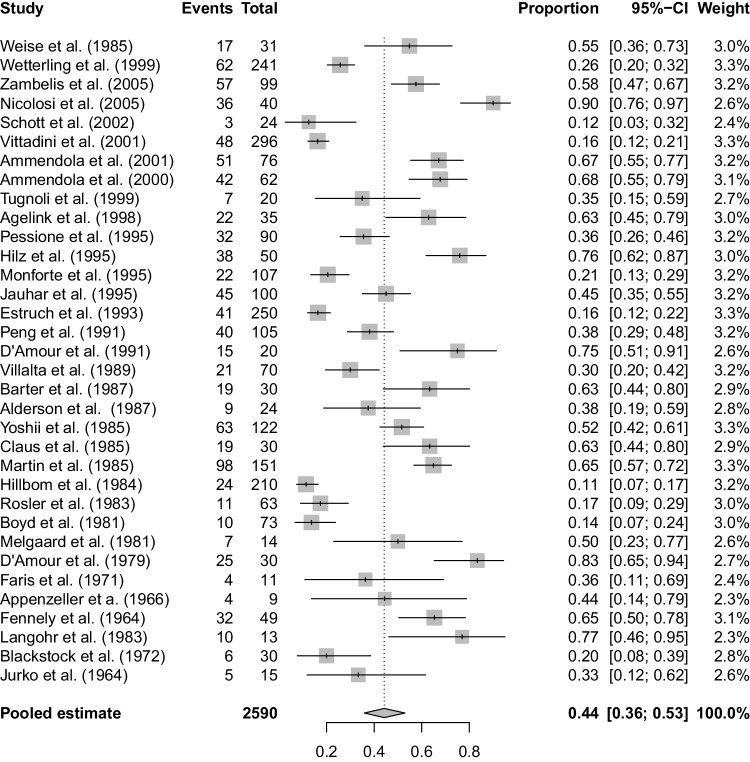
Forest plot for the prevalence of peripheral neuropathy amongst chronic alcohol abusers, diagnosed using history and examination alone

**Fig. 3:**
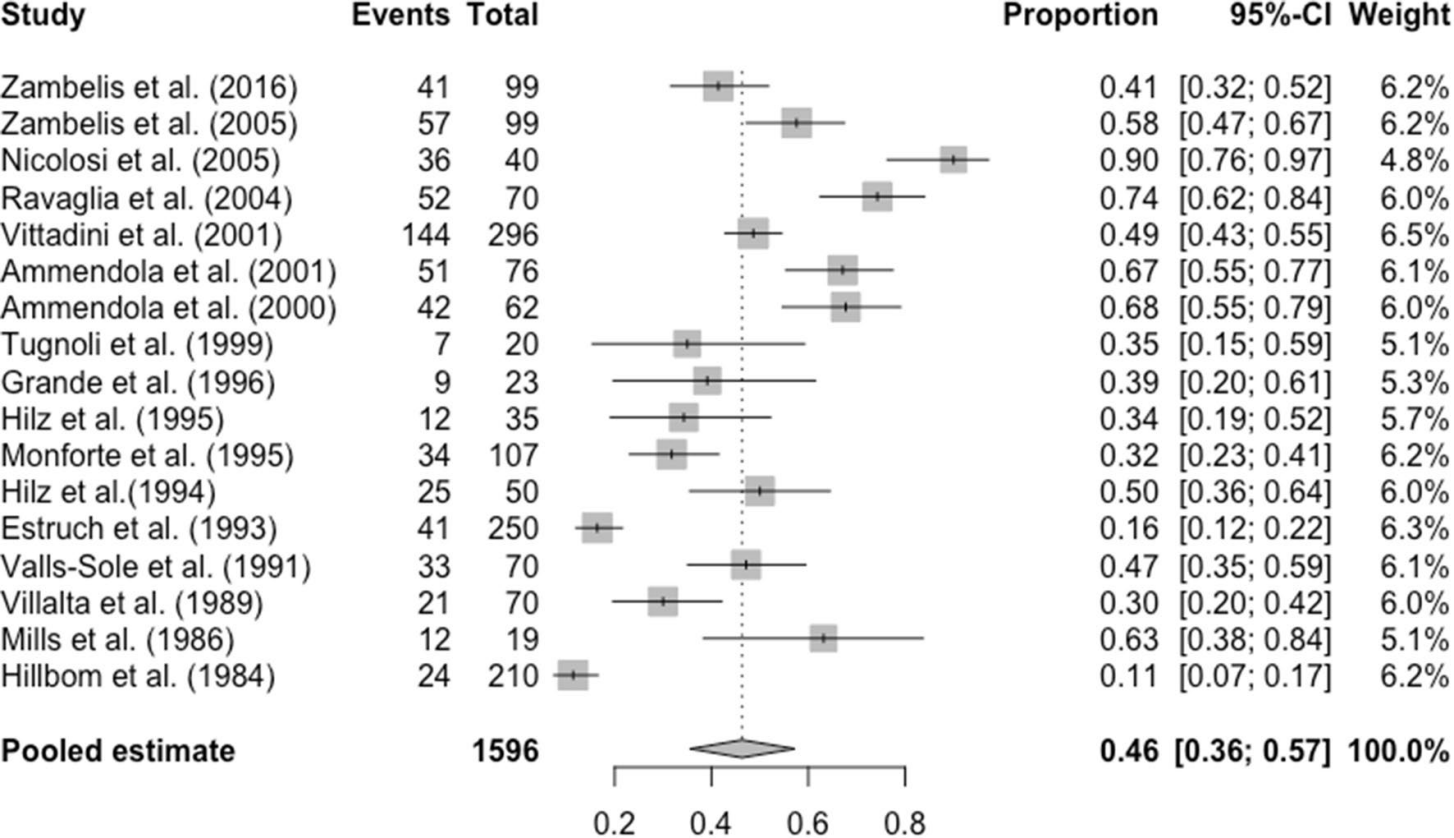
Forest plot for the prevalence of peripheral neuropathy amongst chronic alcohol abusers, diagnosed using nerve conduction studies with or without supplementary electromyography

41 studies investigated a non-selected population of chronic alcohol abusers for peripheral neuropathy by either clinical examination and history and/or electrophysiology including nerve conduction studies (NCS) with or without supplementary electromyogram (EMG) with a focus upon large fibre neuropathy. As seen in Fig. 2, 34 of the studies provided data regarding the prevalence of peripheral neuropathy diagnosed using history and examination, giving a pooled prevalence of 44.2% (CI 35.9–53%, *n* = 2590) [5, 6, 10–40]. As seen in Fig. 3, 17 of the studies gave prevalence data in populations diagnosed peripheral neuropathy using NCS, giving a pooled prevalence of 46.3% (CI 35.7–57.3%; *n* = 1596) [2, 5, 6, 12, 14, 23, 35, 37–39, 41–47]. Just one study focussed on the prevalence of small fibre neuropathy, in which amongst 98 patients, 45 patients had large fibre peripheral neuropathy and 37 patients (37.8%) had small fibre neuropathy [5]. The drawback of this study, however, is that the diagnosis of small fibre neuropathy was only made via QST, which is a psychophysical test, and therefore, prone to biases. Amongst the 45 with large fibre neuropathy, 20 (44.4%) also had small fibre neuropathy.

Based on these studies, it can be determined that there is a high rate of peripheral neuropathy amongst chronic alcohol abusers. It also appears that the addition of NCS may improve the identification of alcohol-related peripheral neuropathy.

#### Prevalence of alcohol-related peripheral neuropathy amongst those with polyneuropathy

Mygland investigated the rate of alcohol-related polyneuropathy in the general population of Vest-Agder, Norway [48]. Based upon a database of 192 polyneuropathy diagnoses made in the country between June 1994 and October 1999 the prevalence of alcohol-related neuropathy was 12.2/100,000 and it represented 10% of polyneuropathies in the region. A study in Taiwan conducted by Lin et al. investigated the aetiology of 520 cases of “generalised neuropathy”, defined as peripheral neuropathy which affected more than one area such as polyneuropathy, multiple mononeuropathies and mononeuritis multiplex [49]. Of this population, 8.7% were identified to have a neuropathy with an alcohol-related cause. Verghese et al. studied the causes of polyneuropathy in an older population of the over 65 (*n* = 402) [50]. Alcohol-related neuropathy represented a decreasing proportion of cases with advancing age, as the cause of neuropathy in 6.1% of those aged 65–75, 1.4% of those aged 75–84, and none of those aged 85 or above.

### Natural history

#### Clinical presentation

**Fig. 4:**
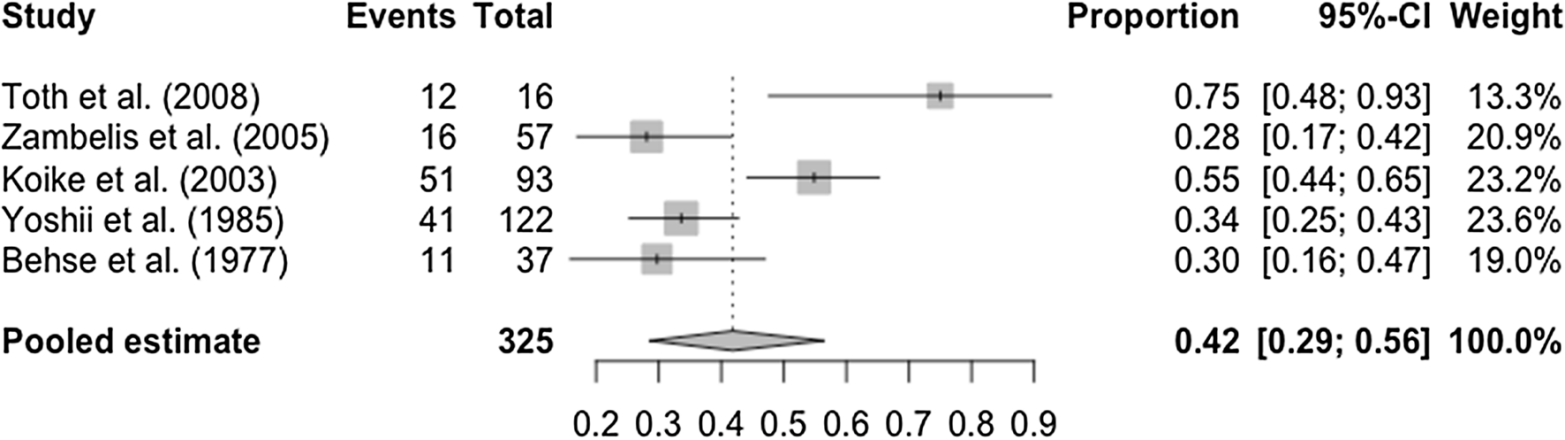
Forest plot for the prevalence of pain amongst individuals with alcohol-related peripheral neuropathy

Alcohol-related peripheral neuropathy has a slow, progressive onset over months to years [3, 4, 51, 52], almost always affects the lower limbs more than the upper limbs and begins distally [2–4, 14, 19, 20, 26, 27, 30, 35, 37–41, 47, 51, 53–59]. Generally, patients present with primarily sensory features, including paraesthesia, numbness and impaired vibration sensation [2, 3, 6, 14, 15, 19, 20, 26, 27, 30, 32, 33, 35, 37–41, 51, 53–56, 60, 61]. Reduced proprioception has been reported in a smaller number of cases [39, 40]. Motor features also occur, most often weakness, but this represented a less frequent finding and very rarely involves the upper limbs [6, 14, 19, 30, 35, 40, 53, 55, 56]. Diminished or absent reflexes are a frequent finding [2, 3, 15, 19, 20, 30, 32, 37–39, 41, 53, 54, 59]. A minority of studies reported painful neuropathies amongst patients, but this was not a feature in all studies and there was significant variation between those which provided data. The prevalence of pain in alcohol-related neuropathy was reported on in 5 studies, and, as seen in Fig. 4, the pooled prevalence was 42% (CI 29–56%, *n* = 325) [3, 5, 19, 59, 62].

#### Nerve conduction studies and electromyography

Thirty studies performed nerve conduction studies. In general, the nerves in lower limbs were more affected than the upper limbs [3, 37–39]. Four studies reported abnormalities only in sensory nerves [33, 47, 63, 64], while ten reported abnormalities in both sensory and motor nerves [2–4, 16, 38, 54, 56, 58, 59, 65]. This may be a reflection of the severity of the neuropathy in which motor nerve function is affected at a later stage. The abnormalities were usually of reduced amplitude, in keeping with axonal loss [2, 3, 5, 11, 12, 16, 21, 27, 37–39, 47, 51, 53, 54, 56, 63–68]. H and F wave latencies were not routinely reported but were found to be prolonged in those with alcohol-related peripheral neuropathy in studies that did [4, 67]. Particular attention was paid to radial SNAPs, tibial CMAPs, and sural SNAPs due to them being spared in entrapment neuropathies unlike the median, ulnar, and peroneal nerves. The sural nerve was the most commonly reported nerve [2, 3, 5, 11, 27, 37–39, 51, 53, 59, 63, 68]. Motor function of the tibial nerve was the next common [3, 11, 51, 54, 59, 63]. Only one study examined radial nerves which reported reduced SNAP [53]. Finally, one study examined the strength-duration time constant (SDTC) and rheobase in median nerves of those with alcoholic peripheral neuropathy [69]. The SDTC was normal compared to controls, but the rheobase was significantly different suggesting that APN may affect internodal channels other than nodal channels or the Na+ –K+ ATP pump.

Nine studies reported EMG findings in alcohol-related peripheral neuropathy patients. Reduced recruitment pattern of motor units was a frequently reported outcome [16, 28, 67, 70]. Active denervation (presence of positive waves and fibrillations) was also present in the majority of patients. The prevalence of denervation findings on EMG ranged from muscle to muscle, with the highest being in the muscles of the lower limbs suggesting a length-dependent pattern [35, 45, 52, 59]. However, one study reported normal EMG findings, which may reflect the early stages of the neuropathy in which predominantly the sensory NCS are abnormal [47] Some studies performed single-fibre EMG which reported increased fibre density in alcoholic patients, suggesting higher re-innervation rates in such patients [52, 70].

#### Risk factors

##### Alcohol intake

Unsurprisingly, intake of alcohol has been positively correlated with prevalence of neuropathy by several authors. Wetterling et al. investigated the prevalence of peripheral neuropathy amongst chronic alcoholics (*n* = 242), splitting the cohort by drinking pattern into episodic drinkers [less frequent, irregular alcohol consumption with longer (> 5 days) sober periods, and some binges (less than one/week)], frequent heavy drinkers [frequent alcohol consumption (more than 3 days/week)] with frequent intoxication (more than one/week) and continuous drinkers (almost daily alcohol consumption without bingeing) [31]. This study demonstrated higher rates of peripheral neuropathy amongst continuous and frequent heavy drinkers (29.6 and 29.9% respectively) than episodic drinkers (11.3%). Similarly, a study by Vittadini et al. (*n* = 296) found duration of alcohol abuse to be amongst the most important risk factors for peripheral neuropathy showing that subjective symptoms developed after a relatively short duration of abuse (1–5 years) but severe polyneuropathy after > 10 years of alcohol abuse. Ammendola et al. compared alcoholics with and without neuropathy to identify risk factors this study showed an increased duration of alcoholism amongst those with neuropathy as well as a higher total lifetime dose of ethanol (TLDE) (*n* = 76) [6]. It also identified an inverse relationship between TLDE and duration of alcoholism and sural nerve SEP amplitude. TLDE was a common factor identified in six further studies which found it to be correlated with an increasing frequency of neuropathy [10, 12, 14, 37, 38, 40]. A study conducted by Angelink et al. also reported a correlation between neuropathy and duration of alcohol abuse; as well as neuropathy and increasing age (*n* = 35) [40], and one study conducted by Pessione et al. found that severity of alcoholism, TLDE and presence of other alcohol-related diseases were significantly related to the presence of neuropathy (*n* = 90) [10]. Conflicting these, two studies were unable to find a relationship between TLDE and neuropathy, though they had reasonably small populations (*n* = 17 and *n* = 46) [66, 71].

##### Sex

Some authors have identified significant relationships between sex and risk of alcohol-related neuropathy. Several studies, including a larger study by Vittadini et al., have found that there is an increased prevalence amongst males [5, 6, 40]. Behse and colleagues, however, found that females were more vulnerable to severe neuropathy, whilst males were overrepresented amongst mild cases, though this is based on a small study (*n* = 37). These studies did not adjust for alcohol consumption, and therefore, this may be because male subjects consume more alcohol as opposed to any biological vulnerability to alcoholic neuropathy in the male sex.

##### Genetics

Rosler et al. investigated the association between HLA distribution and the physical consequences of alcoholism, including polyneuropathy, and showed that there was no relationship between alcohol-related neuropathy and a particular HLA type (*n* = 63) [24]. Masaki and colleagues investigated the role of Glu-487→Lys mutation (single nucleotide polymorphism) of the aldehyde dehydrogenase-2 (ALDH2) in alcohol-related polyneuropathy in a cohort in Japan [72]. The ALDH2*2 mutant allele is inactive, which causes accumulation of acetaldehyde which is thought to be toxic. This study compared 21 alcoholic patients with ALDH2*1/2*1 to 21 alcoholic patients with ALDH2*2/2*1. The study identified that the sensory nerve action potential amplitudes (SNAPs) of the sural and median nerves were significantly lower in the ALDH2*2 heterozygotes than in the ALDH2*1 homozygotes. This, therefore, could be a significant risk factor for alcohol-related neuropathy and also demonstrates that acetaldehyde toxicity may be important in alcohol-related neuropathy. However, it is important to note that although the ALDH*2 allele is prevalent amongst East Asians (and responsible for the well-known “Asian alcohol flushing syndrome”), it is essentially absent amongst Europeans, and therefore, this specific genetic risk factor is population-specific.

Family history has been implicated as a risk factor for alcoholic neuropathy. Ammendola et al. identified a larger proportion of those who abuse alcohol with neuropathy had a family history of alcoholism than those who did not have neuropathy [38]. Similarly, Pessione et al. found a significant relationship between parental history of alcoholism and presence of neuropathy [10]. The association between family history and neuropathy was quite striking, with four times the number of patients with neuropathy having a parental history of alcoholism, compared to those without. It is unclear whether this is a consequence of inherited or environmental risk factors, though the authors suggest that it is possible that there is an inherited genetic risk of developing neuropathy or associated environmental factors.

##### Alcohol type consumed

Uniquely, Vittadini and colleagues found a relationship between the type of alcohol consumed and neuropathy. Specifically, the study demonstrated worse NCS study dysfunction amongst wine drinkers, than those who drank beer or spirits alone [6]. The authors point out that this could be an anomaly due to the wine drinkers consuming more ethanol than other alcohol abusers but offer an alternative explanation that wine may contain more toxic impurities than other beverages. This aspect was not discussed in any other studies.

#### The role of malnutrition

Malnutrition has been implicated in the pathology of alcohol-related neuropathy by several authors. The data, however, is conflicting as to the role which malnutrition plays. The majority of studies which investigate the relationship between malnutrition and neuropathy focus on thiamine deficiency as an aetiological factor, drawing upon existing knowledge of Beri Beri. For the most part, the available literature indicates that alcohol-related neuropathy may occur in the absence of nutritional deficiency, and that neither the prevalence nor the severity of alcohol-related peripheral neuropathy are correlated with nutritional status [3, 5, 14–16, 21, 32, 37, 38, 46, 47, 51, 59, 73–75]. A smaller number of publications do attribute thiamine deficiency, but generally speaking these studies were older or of lower quality evidence [4, 6, 30, 58, 76, 77]. An alternative explanation is that comorbid nutritional deficiency in the context of alcohol-related neuropathy may either increase the risk of neuropathy or that thiamine deficiency neuropathy is often superimposed upon neuropathy caused by the toxic effects of alcohol or its metabolites.

An interesting study by Koike et al. compared the clinical and pathological features of patients with thiamine deficient neuropathy; alcoholic neuropathy without thiamine deficiency and alcoholic neuropathy with thiamine deficiency [3]. The study showed that alcohol-related neuropathy and thiamine deficient neuropathy are clinically and pathologically distinct. The study also showed that the clinicopathological features of alcoholic neuropathy are quite uniform, but that variation occurs with concomitant thiamine deficiency. Specifically, alcohol-related neuropathy presented with slowly progressive, sensory-dominant symptoms whilst thiamine deficiency caused acutely progressive (< 1 month in 56%) primarily motor-dominant features with loss of ambulation, although there was more variation and presentations were inclusive of sensory-dominant cases. On sural nerve biopsy, alcoholic neuropathy showed largely small fibre loss, more frequent myelin irregularly and segmental demyelination/remyelination whilst thiamine deficiency neuropathy showed more large fibre loss and more subperineural oedema. In the patient group with both thiamine deficiency and alcohol excess, the presentations and biopsies included features from across this spectrum of clinicopathological features. The authors determine that the current confusion surrounding the role of nutrition in alcohol-related neuropathy are a consequence of undetected thiamine deficiency in some series (and therefore, variation in the features of patients) and excessive emphasis of the role of malnutrition in others. They also point out that as highly sensitive measurement of thiamine levels in the form of liquid chromatography did not become widely available until the 1980s, and therefore, some author’s assessments of nutritional status may have been inadequate.

#### The role of hepatic dysfunction

An association between chronic hepatic dysfunction and neuropathy has been noted by several authors [78]. This has led some authors speculate that hepatic dysfunction, most often cirrhosis, may be important to the pathogenesis of alcoholic peripheral neuropathy. Zambellis et al. (*n* = 99) found that polyneuropathy amongst alcohol abusers was significantly correlated with liver dysfunction [5]. Vittadini et al. (*n* = 296) found a significant correlation between liver disease and the severity of polyneuropathy amongst chronic alcohol abusers [6]. Conversely, other authors have failed to find any significant relationship between hepatic dysfunction and neuropathy (*n* = 383) [14, 79].

Two small studies compared the rates of peripheral neuropathy between those with alcoholic liver disease and those without alcoholic liver disease to establish the importance of each element. Thuluvath and Triger found that 45% of those with alcoholic liver disease and 22% with non-alcoholic liver disease had peripheral neuropathy (*n* = 64) [74]. Kharbanda et al. compared patients with alcohol-related cirrhosis to those with non-alcoholic cirrhosis (*n* = 33) finding that incidence of neuropathy was 88% in alcoholic cirrhosis compared with 56% in non-alcoholic cirrhosis [80]. This difference did not reach significance, leading the author to judge that it was the hepatic dysfunction which was most important to cause the neuropathy. However, as this study is small, it does not appear to have the strength to draw such a conclusion. These studies illustrate that hepatic dysfunction is in itself a cause of neuropathy, and that it may account for some cases of alcohol-related neuropathy.

Knill-Jones et al. studied a small group of 14 individuals who had both peripheral neuropathy and hepatic disease in the form of cirrhosis or hepatitis. Four of this group had alcoholic cirrhosis [81]. Sural nerve biopsies of those with hepatic disease of alcoholic aetiology and non-alcoholic aetiology were similar. Likewise, in a cohort of chronic alcohol abusers with peripheral neuropathy, Behse et al. found that histological abnormalities were unrelated to the presence or absence of liver disease (*n* = 37) [59].

The available data addressing the role of hepatic dysfunction is presently inconclusive. It is possible that hepatic dysfunction and alcoholic toxicity each cause neuropathy independently, and that there is frequently overlap between the two. It may also be that comorbid hepatic dysfunction is a risk factor for alcohol-related peripheral neuropathy. Further studies are required to develop a greater understanding of the interaction these entities.

### Biopsy results

Thirteen studies provided data from the biopsy of the sural nerve or the skin in patients with alcohol-related peripheral neuropathy. This data, while validated was at times incongruent. Within these studies, morphological heterogeneity has been extensively reported, and axonal degeneration is reported as the primary pathology for neuropathies in the vast majority of the studies, with a minority describing findings of demyelination and remyelination [3, 51, 53, 56, 59, 82–84]. Alcohol-related peripheral neuropathy appears to be characterised by severe loss of myelinated fibres; and although profound small fibre loss can also be present, this appears to occur more variably [3, 51, 53, 59, 85]. The data indicates that there is both small and large fibre loss in alcohol-related neuropathy, but that small fibre loss is generally predominant [3, 51, 53, 56, 59, 63, 86]. Koike et al. assessed the sural nerve biopsy results in 18 patients with painful alcohol-related polyneuropathy and found that while those with a shorter disease duration had predominantly small myelinated fibre loss, those with a course greater than 5 years had comparatively abundant small fibres with more large fibre involvement [51].

Epidermal nerve fibre density was assessed in two studies, both of which supported decremental nerve fibre density distally in the lower limb, anecdotally supportive of a length-dependent pattern [53, 63]. The sometimes-conflicting findings between biopsy findings may be representative of the complex interplay of pathological factors in alcohol-related peripheral neuropathy and is indicative of the need for further research in this area.

### The role of inflammation

A minority of studies also looked at pathophysiology through cell immunohistochemistry of biopsy samples and histological analysis of blood. Michałowska-Wender et al. measured inflammatory cytokines in the sera of patients with alcohol-related neuropathy (*n* = 31) and controls [87]. Specifically, the authors measured tumour necrosis factor alpha (TNF-α), monocyte chemotactic protein-1 (MCP-1) and growth-regulated protein alpha (GRO-α). Neither TNF-α nor MCP-1 differed significantly between patient and control sera. GRO-α, however, was significantly higher amongst those with alcohol-related polyneuropathy. The role of GRO-α is unclear, and therefore, this does little to illuminate the pathogenesis of alcohol-related neuropathy. It is known to be involved in both primary and secondary inflammatory reactions, is a chemotactic agent for neutrophils and is involved in tumorigenesis, but its specific relevance in alcoholic neuropathy is unclear.

### The role of oxidative stress

Haslbeck et al. used immunohistochemistry to study the localisation of N^ε^-Carboxymethyllysine (CML), a marker of cumulative oxidative stress, in the sural nerves of patients with various polyneuropathies (*n* = 31) [82]. CML was identified in the perineurium of the sural nerves of those with alcohol-related polyneuropathy (*n* = 4), which may indicate dysfunction in this aspect of the nerve. The authors speculate that as the perineurium is thought to have a barrier function, that this could cause pathological changes in the endoneurial environment. There are currently no studies to support or enrich these findings, highlighting a need for further research into the pathophysiological processes.

### Management

Four studies addressed the management of patients with alcohol-related peripheral neuropathy. These studies addressed abstinence from alcohol consumption and administration of vitamins.

#### Abstinence

Hawley et al. followed up 11 patients with alcohol-related neuropathy who were abstinent from alcohol and who had begun to consume a normal diet [67]. This identified improvement in sensory symptoms within a few days and a clinical improvement in strength over a period of weeks to months, but in up to 2 years in the most severe cases. There was not however, complete resolution of symmetric neuropathy with persistent mild loss of vibration sense or pinprick sensation in the feet or loss of ankle tendon reflexes.

#### Vitamins

Peters et al. conducted a multicentre, randomised, double-blind placebo-controlled study in which they compared two B vitamin formulations for the treatment of alcohol-related polyneuropathy with sensory symptoms and signs (*n* = 253) [88]. This study compared a formulation containing vitamins B1, B2, B6 and B12, and a new formulation contains these with the addition of B9 (folic acid). Patients were followed up at 6 and 12 weeks following the beginning of treatment. At 12 weeks follow up, both of these formulations showed efficacy compared with placebo. There was a significant improvement in two-point discrimination, great toe vibration sensation and pain intensity at both 6 and 12 weeks. There was also an improvement in eye-nose coordination and knee jerk and Achilles tendon reflex responses (scored as ‘increased’, ‘normal’, ‘decreased’, or ‘absent’) in 12 weeks in both groups compared with placebo. There were not significant differences between the new and old formulations. This study demonstrated that such formulations were efficacious to manage alcoholic polyneuropathy, but that the addition of folic acid had no significant benefit.

Woelk et al. conducted a three armed, randomised, double-blind, placebo-controlled trial in which they compared benfotiamine; a combination of neurotropic B vitamins and benfotiamine; and placebo in the treatment of alcohol-related polyneuropathy (*n* = 84) [60]. Treatment lasted 8 weeks with a total of five examinations at 2-week intervals. Peripheral nerve function was assessed with vibration perception thresholds, intensity of pain with McGills pain questionnaire, assessment of paralysis, distal touch sensation, coordination, and tendon reflex impairment. This study showed no statistically significant effect in the benfotiamine + neurotropic B vitamin group relative to placebo. The group receiving benfotiamine alone were significantly improved in terms of great toe vibration sensation, motor function, and overall neuropathy score, as well as this there were no adverse events. This study demonstrated efficacy for benfotiamine, but it is unclear why the combined treatment group did not have significantly better outcomes, which brings the results of this trial into question.

Fennelly and colleagues evaluated the response to vitamin therapy in 29 individuals with alcohol-related neuropathy [30]. Patients were admitted and treated with a diet containing thiamine, nicotinic acid, pantothenic acid, pyridoxine, folic acid, and vitamin B12. Additionally, patients received intramuscular injections of thiamine. This study found that the response to treatment depended upon the severity of neuropathy and whether there was severe cirrhosis. Thiamine replacement improved signs of neuropathy in 7/13 patients with grade I neuropathy (objective signs of reduced pain and vibration sensation but normal reflexes) and 3/8 with grade II (marked sensory changes and decreased reflexes) neuropathy within 4 weeks. No patients with grade III (severe sensory impairment, absent reflexes, foot drop, muscle wasting) neuropathy showed clinical improvement over the 4-week period, but 4/8 did show an improvement over 3–6 months. Amongst those who did not respond to thiamine, two patients with grade I neuropathy and one with grade II responded with the correction of low circulating nicotinic acid. One patient with grade I neuropathy responded with the correction of low pantothenic acid. One patient with grade III neuropathy responded with the correction of low circulating vitamin B6. This study showed that as well as thiamine replacement, corrections of low circulating levels of nicotinic acid, pantothenic acid and vitamin B6 can result in an improvement of alcohol-related peripheral neuropathies.

Based upon these results, vitamin supplementation appears to exert a positive therapeutic effect in alcohol-related neuropathy. The mechanism of this is presently unclear, one possible explanation is that is resolves concomitant vitamin-dependent neuropathy which exacerbates alcohol-related neuropathy.

### Assessment of bias

For the most part this review consists of non-interventional studies for which generally accepted tools to evaluate risk of bias are not available. However, bias was still considered when evaluating studies as these study types were subject to the following limitations; population selection bias, loss of patients at follow ups, bias through misclassification or misdiagnosis, patient recall and observer bias. A minority of included studies were interventional RCTs. To assess the bias in these we applied the Jadad score which takes into consideration quality of randomisation and blinding as well as reporting of withdrawals to assess bias in RCTs [9]. All RCTs that were included As well as this, where interventional studies are cited a clear description of their design is in text to allow the reader to evaluate that articles risk of bias.

## Conclusions

In summary, the present study makes the following conclusions regarding alcohol-related peripheral neuropathy:

- Alcohol-related peripheral neuropathy is common, with signs and symptoms in 44% of chronic alcohol abusers and representing 10% of polyneuropathies. When utilising NCS to identify subclinical neuropathy amongst alcohol abusers, the rate is higher.
- The pooled prevalence of pain amongst alcoholic neuropathy sufferers is 42%. Although this figure should be interpreted with caution as it is based on a small number of studies, it suggests that alcohol-related neuropathy is one of the least painful neuropathies [89–93]. There is a need for more careful mapping and description of the symptoms of neuropathy in research and clinical practice.
- Alcohol-related peripheral neuropathy is primarily an axonal, length-dependent, sensorimotor neuropathy with dominant sensory features.
- TLDE is currently the best validated risk factor for development of alcohol-related peripheral neuropathy. Other risk factors include pattern of alcohol consumption, parental history of alcohol abuse, male gender, and mutation of *ALDH2*.
- Some authors identify hepatic dysfunction and malnutrition, particularly of thiamine, to be central to the pathological process in alcohol-related neuropathy. The evidence presented in this review may suggest that this is not that case, but that these might represent additional risk factors or perhaps cause neuropathy independently which is superimposed upon that caused by the neurotoxic effects of alcohol. However, the relationship between ethanol toxicity and neuropathy is as of yet unproven and there are other possible risk factors not yet addressed by the literature. To give one such example, there is a well-documented association between smoking tobacco and alcoholism, and smoking has been associated with increased risk of peripheral neuropathy in other populations such as in those with diabetes [94, 95]. Further studies are required to investigate the relationships between these factors and to translationally explore the relationship between ethanol consumption and neuropathy.
- There is presently little literature regarding the optimal management strategy for those with alcohol-related neuropathy. Vitamin supplementation appears to currently have the best evidence.
- Although NCS are better in determining the presence of peripheral neuropathy and its severity, they were not used consistently in all studies. Additionally, the neurophysiological parameters used were inconsistent. Routine use of NCS of lower and upper limbs in the diagnosis of PN in patients is advised.
- There is currently very little data focussed upon the prevalence of small fibre neuropathy in the context of alcoholic neuropathy. This should be a focus of future research.

## Limitations

- There was a great deal of heterogeneity between studies with respect to the definitions of alcohol abuse and the means used to diagnose peripheral neuropathy. Alcohol misuse/addiction was defined in a number of ways, sometimes using validated criteria such as diagnostic and statistical manual of mental disorders (DSM), and sometimes arbitrarily. With regards to neuropathy, the present study aimed to be clear whether patients were diagnosed based upon clinical features, nerve conduction studies or both when discussing prevalence. The variation in definitions of alcohol abuse, however, was not resolvable, though this review removed patients who were not chronic abusers and aimed to draw distinctions between duration of alcohol consumption and duration of abuse between studies where possible.
- There were no restrictions in date of publication applied in this review. This was a deliberate decision made to review the full range of literature pertinent to the topic in question. However, as the included literature dates as far back as 1964 it is feasible that modern investigations may have identified causes of neuropathy other than alcoholism amongst some included patients. Another consequence of inclusion of older literature is heterogeneity in the means used to measure thiamine deficiency, with some studies having used the less accurate, indirect approach of erythrocyte transketolase activity assay to discern thiamine status.
- Nutrition and liver function were not addressed in all studies. Therefore, in some studies it cannot be certain to what degree the neurological dysfunction is a consequence of alcohol toxicity.
